# Multi-omics integration in deciphering non-small cell lung cancer drug resistance: current status, challenges, and future prospects

**DOI:** 10.1186/s41065-025-00570-w

**Published:** 2026-02-02

**Authors:** Jidong Miao, Wenqiang Guan, Jing Wang, Huiying Gong, Qian Xie, Yang Gao

**Affiliations:** 1 Department of Oncology, Zigong Fourth People’s Hospital, Zigong, Sichuan Province 643000 China; 2 School of Education and Sports, Sichuan Vocational College of Health and Rehabilitation, Sichuan Province, Zigong, 643000 China

**Keywords:** Non-small cell lung cancer, Multi-omics integration, Drug resistance mechanism, Proteomics, Genomics, Single-cell analysis

## Abstract

Lung cancer is the leading cause of cancer-related deaths globally. Non-small cell lung cancer (NSCLC) accounts for approximately 85% of all lung cancer cases, and drug resistance severely undermines treatment efficacy. This review summarizes recent advances in elucidating NSCLC drug-resistance mechanisms using multi-omics integration. Multi-omics integration systematically reveals the molecular networks of drug resistance, identifies key biomarkers and targets, and facilitates the screening of high-priority candidates for drug development through experimental validation. Small-molecule inhibitors targeting drug-resistant proteins and multi-omics-guided combination therapies offer strategies to reverse resistance. Future directions involve developing simultaneous multi-omics detection technologies, leveraging artificial intelligence for intelligent data analysis, establishing standardized frameworks for data sharing, and implementing personalized medicine based on multi-omics to improve patient prognosis.

## Introduction

### Background and significance of NSCLC

Lung cancer is the most common cause of cancer-related mortality globally [52]. Non-small cell lung cancer (NSCLC) represents the predominant histological subtype [46, 70]. Although considerable progress has been made in NSCLC treatment—evolving from surgery, chemotherapy, and radiotherapy to molecularly targeted therapy and immunotherapy—the five-year survival rate remains poor [92].

The development of drug resistance constitutes a major obstacle in NSCLC management [81]. Regardless of the treatment modality, whether chemotherapy or targeted agents, most patients eventually develop resistance, which diminishes therapeutic efficacy and often leads to disease recurrence or progression [30, 140]. Conventional research into resistance mechanisms has primarily focused on a single dimension, such as genetic mutations. However, tumor drug resistance is a complex biological process involving dysregulation across multiple molecular layers and pathways. Consequently, studies relying on a single omics dimension are insufficient for comprehensively elucidating these mechanisms [31].

Proteomics, which involves the large-scale study of proteins in cells, tissues, or organisms, provides direct insight into cellular functional states under specific physiological or pathological conditions [8, 99]. Recent advances in proteomic technologies have offered new perspectives and tools investigating for NSCLC drug resistance [77]. In parallel, the concept of multi-omics integration has gained prominence. approach integrates proteomics with various omics technologies such as genomics, transcriptomics, and metabolomics [126]. Integrating data across these layers provides new opportunities to deepen our understanding of resistance mechanisms in NSCLC.

This review aims to synthesize recent progress in proteomics-based multi-omics integration for deciphering drug resistance mechanisms in NSCLC. A key emphasis is placed on the integrative role of proteomics in linking genomic and transcriptomic alterations with functional protein-level changes that drive resistance—a perspective that distinguishes this review from existing works that treat proteomics in isolation or multi-omics only generically. We propose that a proteomics-centered integrative framework can more effectively reveal multi-dimensional molecular networks underlying resistance, which may be overlooked in single-omics or non-integrated multi-omics analyses. Additionally, this review discusses translational applications of these insights, from drug target discovery to strategies for overcoming resistance, thereby providing a theoretical foundation and novel perspectives for future precision medicine in NSCLC.

### Overview of multi-omics technologies

#### Proteomics

Proteomics refers to the large-scale study of protein characteristics, including expression levels, post-translational modifications (PTMs), and protein–protein interactions, providing comprehensive insight into cellular functions and disease processes at a functional level [11]. Mass spectrometry (MS) serves as the cornerstone technology, enabling the separation, identification, and quantification of proteins to analyze their abundance, modification status, and interactions systematically [38, 149]. In NSCLC research, proteomics plays a pivotal role in characterizing functional cellular states and identifying key proteins involved in tumorigenesis, progression, and drug resistance [144]. For instance, the study of KRAS mutations, a key driver in lung cancer, has been advanced through proteomics [49, 137]. Researchers employed TMT quantitative proteomics to investigate resistance mechanisms of novel KRASG12C inhibitors, creating an extensive proteomic library of KRAS inhibitor responses. This study elucidated mechanisms of resistance and identified potential combination therapies aimed at improving the clinical efficacy of KRASG12C inhibitors [108]. Two primary technological strategies are widely employed. Liquid chromatography-tandem mass spectrometry (LC–MS/MS)-based methods, such as shotgun proteomics, allow large-scale identification and quantification of proteins and their PTMs like phosphorylation and ubiquitination, which are crucial for understanding signaling networks in NSCLC resistance [88, 139]. For example, proteogenomic analyses have identified molecular subtypes with distinct immune features and potential therapeutic targets [58, 69]. Stable isotope labeling techniques (e.g., SILAC, iTRAQ, TMT) enable precise comparison of protein expression between drug-resistant and sensitive cells, and facilitate PTM analysis to uncover regulatory mechanisms [23, 67]. Antibody-based approaches, including antibody arrays and immunoprecipitation (IP) coupled with MS, utilize specific antigen–antibody interactions for high-throughput protein profiling and interaction network mapping. These methods are valuable for screening for diagnostic or prognostic biomarkers and in elucidating key protein complexes and signaling pathways. For instance, IP-MS analysis revealed that USP7 binds to and stabilizes KRAS-G12C, suggesting a promising combinatorial therapeutic strategy to tackle resistance [9, 19–21, 120].

#### Genomics

Genomics involves the comprehensive analysis of an organism’s complete DNA sequence, encompassing mutations, structural variations, and gene amplifications or deletions through techniques such as whole-genome sequencing (WGS) and genotyping [43, 82, 119]. In NSCLC, genomics has played a fundamental role in identifying oncogenic driver mutations (e.g., in EGFR, ALK, and KRAS), which have transformed targeted treatment approaches [10]. Beyond initial diagnosis, genomic profiling offers crucial insights into resistance mechanisms, often involving secondary mutations in the primary driver gene (e.g., EGFR T790M/C797S) or activation of bypass signaling pathways (e.g., MET amplification) [25, 48]. Recent advances in understanding tumor evolutionary dynamics have been exemplified by studies such as TRACERx (Tracking Cancer Evolution through Therapy Rx). The 2023 TRACERx update Analyzed 1,644 tumor regions from 421 patients with NSCLC and revealed that intratumor heterogeneity (ITH) is a key determinant of metastatic spread and treatment failure. Notably, spatial genomic profiling showed subclonal expansions within hypoxic niches enriched for resistance-associated mutations [85]. Moreover, the integration of ​​liquid biopsy analyses demonstrated that preoperative ctDNA negativity in lung adenocarcinoma patients was associated with superior overall survival (OS): the favorable OS rate was 59% in ctDNA-negative patients [1]. These findings highlight the value of genomic profiling in elucidating resistance evolution and guiding precision oncology strategies.

#### Transcriptomics

Transcriptomics involves the comprehensive profiling of RNA transcripts within a cell or tissue, primarily through RNA sequencing (RNA-seq) [32]. It enables quantification of gene expression levels, discovery of novel transcripts, and analysis of alternative splicing events [133]. In NSCLC drug resistance, transcriptomics studies capture large-scale dynamic changes in gene expression programs. For example, research has shown that ferroptosis-related genes (such as TFR1, FPN, SLC7A11, and GPX4) are dysregulated in osimertinib-resistant cells, suggesting a potential link between ferroptosis evasion and acquired resistance [13]. Beyond bulk RNA-seq, single-cell RNA-seq (scRNA-seq) dissects transcriptional heterogeneity within tumors, identifying rare resistant subpopulations and characterizing diverse cell states in the tumor microenvironment (TME), including immune cell populations and stromal compartments, uncovering transcriptional programs associated with therapy resistance [74, 135]. Recent applications of scRNA-seq And spatial transcriptomics in studies of neoadjuvant immunochemotherapy for NSCLC have further elucidated mechanisms of treatment response. In one study involving 12 patients And 16 tumor samples, integrated single-cell and spatial transcriptomic analyses identified specific immune cell subsets as predictive biomarkers of sensitivity [28]. Notably, SELENOP-expressing macrophages were found to interact synergistically with cancer-associated fibroblasts (CAFs), and their recruitment patterns and crosstalk with T cells were delineated, offering insights into TME remodeling during combination therapy. Spatial transcriptomics enhances these findings by mapping gene expression to histological locations, thereby preserving spatial context often lost in single-cell suspensions. This approach helps identify region-specific resistance mechanisms, such as immune exclusion zones, stromal–epithelial communication, and niche-driven adaptive signaling [78]. In a complementary study of 19 NSCLC patients undergoing immune checkpoint blockade (ICB) therapy, single-cell And spatial transcriptomic profiling of over 232,000 cells revealed that interactions between tumor cells and specific macrophage/CAF subsets hindered T cell infiltration—a feature correlated with poorer prognosis. The same study also identified distinct functional states of tertiary lymphoid structures (TLS) within the TME and associated these with patient outcomes, providing new insights into ICB response mechanisms [146, 147]. Large-scale cohort studies further emphasize the role of transcriptional heterogeneity. The prospective TRACERx study integrated whole-exome And transcriptomic data from 947 tumor regions (primary and metastatic) And 96 normal samples across 354 NSCLC patients. It demonstrated that intratumoral transcriptomic diversity significantly contributes to tumor evolution and exposes heterogeneity not detectable through genomic alterations alone [89].

#### Metabolomics

Metabolomics involves the comprehensive analysis of small-molecule metabolites, which represent the downstream products of genomic, transcriptomic, and proteomic activities. It provides a direct functional readout of cellular states and physiological conditions by quantifying metabolites related to energy production (e.g., glycolysis, TCA cycle), biosynthesis, and signaling pathways [100]. In NSCLC drug resistance, metabolomics has revealed extensive metabolic reprogramming that promotes cancer cell survival under therapy. For example, resistant cells frequently demonstrate enhanced glycolytic flux (the Warburg effect), increased glutaminolysis, and altered lipid metabolism to meet elevated energy and biosynthetic demands during therapeutic stress [94]. These metabolic adaptations are reflected by altered metabolite abundance (e.g., elevated lactate, glutamate, and acetyl-CoA) and are often regulated by corresponding enzymatic upregulation, as detected by proteomics [16]. Integrating metabolomics with other omics layers is therefore essential for understanding how genetic and transcriptional alterations lead to functional metabolic vulnerabilities that may represent therapeutic targets.

#### The significance of multi-omics integration​

Tumor drug resistance is an extremely complex biological process involving aberrant regulation across genomic, transcriptomic, proteomic, and metabolomic levels [125]. Although single-omics approaches provide valuable insights, they are insufficient to fully capture the cascade of molecular events underlying resistance. Multi-omics integration combines complementary data layers to construct a holistic, systems-level perspective, yielding synergistic insights beyond the reach of any single omics method [53, 142]. Each omics layer offers unique information: genomics detects genetic variants linked to resistance,transcriptomics reveals dynamic expression changes during resistance acquisition; proteomics identifies functional alterations in proteins and pathways associated with resistance [73, 84]. By integrating these data, a comprehensive molecular regulatory network of NSCLC drug resistance can be constructed, illustrating how genetic variants affect protein expression through transcriptional regulation and ultimately lead to resistant phenotypes [47]. This systematic strategy allows researchers to trace resistance mechanisms from genetic mutations through transcriptional and proteomic changes, providing a foundation for understanding and overcoming NSCLC drug resistance.

Substantial evidence highlights the power of this approach. One study integrated genomics, transcriptomics, proteomics, phosphoproteomics  and acetylproteomics data from 229 Korean NSCLC patients, identifying five molecular subtypes with distinct clinical and tumor microenvironment (TME) features and elucidating mechanisms of whole-genome duplication [112]. Another investigation employed spatial transcriptomics, single-cell transcriptomics and bulk transcriptomics combined with multiplex fluorescence staining to analyze tumor samples from 19 NSCLC patients before and after neoadjuvant immunotherapy. This integrated multi-omics strategy revealed dynamic remodeling of the TME, identified novel biomarkers, and uncovered previously unrecognized resistance mechanisms related to combined immuno-chemotherapy [146, 147].

## Current understanding of NSCLC drug resistance

The therapeutic drugs for NSCLC primarily include targeted drugs and chemotherapeutic compounds. The resistance mechanisms of these two drug categories differ considerably and profoundly impact clinical efficacy and patient prognosis [152].

### Tyrosine kinase inhibitors (TKIs) and their resistance mechanisms

The efficacy of targeted therapy in advanced NSCLC with sensitive epidermal growth factor receptor (EGFR) mutations or anaplastic lymphoma kinase (ALK) fusions is well-established in clinical practice and is closely linked to molecular subtypes [87, 163]. The positive rate of sensitive EGFR gene mutations is approximately 40–50% among NSCLC patients. epidermal growth factor receptor tyrosine kinase inhibitors (EGFR-TKIs) are standard targeted treatment for stage IV driver-positive NSCLC and are categorized into generations: first-generation (gefitinib, erlotinib, icotinib), second-generation (afatinib, dacomitinib), and third-generation (osimertinib, almonertinib, furmonertinib) agents [121, 131].

Resistance to first-generation EGFR-TKIs is dominated by the secondary EGFR T790M mutation (accounting for about 50% of cases), which increases steric hindrance at the drug-binding site [105, 138]. Additional mechanisms include bypass pathway activation—such as MET amplification (approximately 15%), HER2 amplification, epithelial-mesenchymal transition (EMT), and small cell lung cancer (SCLC) transformation [62]. Second-generation TKIs face similar on-target secondary mutations. Resistance to third-generation EGFR-TKIs, such as osimertinib, is more complex and involves EGFR C797S mutations (cis/trans configurations), AXL kinase activation, BRAF fusions, and immunosuppressive cell infiltration within the TME [117]. Notably, a prospective study utilizing Olink Immuno-Oncology Panel technology Analyzed plasma proteomics from 34 advanced NSCLC patients (with 30 validation cases) And constructed An I-SCORE model via LASSO-Cox regression based on 8 proteins (including CCL23 and ARG1). This model achieved An AUC of 0.94 for predicting 12-month overall survival and revealed associations between soluble immune molecules (e.g., CD28, CXCL10) and T-cell exhaustion—offering novel insights into immune-related osimertinib resistance mechanisms [40]. Resistance to ALK-TKIs (such as crizotinib and alectinib) commonly involves secondary ALK mutations (e.g., L1196M), bypass signaling activation (e.g., EGFR or MET), and tumor heterogeneity [96, 101]. Acquired drug resistance typically emerges within 6–18 months of treatment, while primary drug resistance may result from target-negative disease or pre-existing resistant subclones [57].

### Chemotherapeutic drug and their drug-resistance patterns

Chemotherapy remains a cornerstone for driver-negative NSCLC or post-resistance settings, but its efficacy is limited by diverse resistance mechanisms. Platinum-based drugs (cisplatin, carboplatin) mainly induce apoptosis via DNA cross-linking [5, 60]. Resistance mechanisms include enhanced nucleotide excision repair (NER) (e.g., ERCC1 overexpression), activation of the DNA damage response (DDR) pathways (such as ATM/ATR mutations), and abnormal cell-cycle regulation (such as p53 mutations) [15]. The drug resistance of anti-microtubule agents (paclitaxel, docetaxel) is associated with altered tubulin isotype expression, abnormal PTMs of α/β-tubulin, and overexpression of anti-apoptotic proteins (such as the Bcl-2 family) [103, 122]. For antimetabolites such as pemetrexed, resistance involves downregulation of folate transporters (e.g., SLC19A1), amplification of thymidylate synthase (TS), or epigenetic alterations [76, 80].

Beyond drug-specific mechanisms, a broader challenge is multidrug resistance (MDR), which is driven by factors including ATP-binding cassette (ABC) transporter-mediated drug efflux (e.g., P-glycoprotein [P-gp] and MRP1), enrichment of cancer stem cells (CSCs), and fibrosis-induced impairment of drug delivery in the TME [12, 64]. Recent studies have elucidated regulatory roles of long non-coding RNAs (lncRNAs) in MDR—some lncRNAs sequester microRNAs that repress ABC transporters (e.g., ABCB1 encoding P-gp) or alter epigenetic landscapes, thereby enhancing drug efflux and reducing intracellular drug accumulation [33]. Transcription factors also regulate MDR: forkhead box O1 (FOXO1), a lung cancer tumor suppressor, links to chemo- and radio-resistance via roles in cell cycle arrest and cell death. Its dysfunction impairs drug response in NSCLC, with resistance modulated through DNA repair and oxidative stress pathways; activating FOXO1 or its upstream regulators may reverse resistance [34].

In summary, TKI resistance is largely characterized by on-target mutations and bypass signaling activation, while chemoresistance more commonly involves drug metabolism alterations and cellular stress responses. Although these mechanisms operate through distinct pathways, they often intersect and coexist, collectively leading to treatment failure in NSCLC. Deciphering their interconnected networks using multi-omics technologies is essential to identify targets for precision medicine approaches aimed at reversing resistance [98]. The therapeutic arsenal for NSCLC has expanded to include immunotherapies and cell-based treatments alongside conventional modalities, yet drug resistance remains a major obstacle to improving patient survival. Emerging evidence suggests that resistance to third-generation EGFR-TKIs (e.g., osimertinib) may also involve immune evasion mechanisms, such as reduced CD8^+^ T-cell infiltration and accumulation of immunosuppressive cells (Tregs and MDSCs), highlighting the need for integrated multi-omics and immune profiling [44, 106].

## Multi-omics linkage in NSCLC drug resistance research​

### Integration of proteomics with genomics​

Proteomic research does not operate in isolation. The integration of proteomics with genomics reveals that changes in protein expression are closely associated with genomic alterations such as copy number variations, epigenetic silencing, and changes in mRNA expression [41]. Through this multi-omics correlation analysis, researchers can comprehensively analyze the complex changes from the genetic to the protein level during the process of drug resistance in NSCLC. Genomics identifies mutations, copy number variations, and gene expression profiles, while proteomics elucidates their functional consequences through protein expression and PTMs [83]. This integrated approach enables the linking of genetic alterations to downstream protein-level changes. A prominent example is the study of EGFR T790M-mediated resistance. While genomics detects this acquired mutation, phosphoproteomic analyses demonstrate resultant hyperphosphorylation of downstream effectors such as AKT and ERK, confirming sustained oncogenic signaling [22]. These aberrant phosphorylation patterns serve as critical functional indicators of EGFR-TKI resistance. Furthermore, this integrated understanding directly informs therapeutic development. For instance, the novel inhibitor YK-029A targets both T790M And exon 20 insertion mutations by specifically inhibiting EGFR and its downstream phosphorylation signaling, thereby suppressing tumor growth and promoting apoptosis [79]. The development of YK-029A illustrates how proteomic validation of genomic findings can drive drug innovation. Beyond elucidating targeted therapy resistance, multi-omics integration uncovers novel protein-gene interactions and resistance mechanisms not explainable by genomics alone. For example, although EGFR mutations predict initial TKI sensitivity, subsequent proteomic changes—such as alternative pathway activation or overexpression of drug-efflux proteins—often drive resistance [63, 114]. Integrated multi-omics analysis facilitates the identification of more precise biomarkers and therapeutic targets, advancing personalized treatment strategies for NSCLC patients [68].

### Proteomics-transcriptomics integration​

Proteomics provides an essential complementary perspective to transcriptomic data, particularly in capturing post-transcriptional regulatory events that contribute to drug resistance in NSCLC. Integrated analyses show that while mRNA and protein levels are sometimes correlated—for example, concurrent upregulation of drug-metabolizing enzymes such as glutathione S-transferases in EGFR-TKI-resistant cells enhances drug clearance [110]—they frequently diverge. A notable instance is the tumor suppressor PTEN, whose protein levels are often markedly reduced in resistant cells despite unchanged mRNA abundance, suggesting post-transcriptional regulation through mechanisms such as impaired translation or enhanced degradation that foster treatment tolerance [116, 156]. Non-coding RNAs emerge as key mediators of such discrepancies. Transcriptomic profiling has revealed that miR-34a, which is downregulated in cisplatin-resistant NSCLC, directly targets MYCN—a finding supported by loss- and gain-of-function studies indicating that modulating miR-34a expression influences cisplatin sensitivity [93, 111]. These observations underscore the importance of post-transcriptional regulation, often orchestrated by RNA-binding proteins (RBPs). For instance, the RBP HuR binds to and stabilizes mRNAs encoding anti-apoptotic proteins like Bcl-2, increasing their translation and enabling cancer cells to evade therapy-induced apoptosis [37, 141]. Emerging single-cell multi-omics technologies (e.g., simultaneous measurement of RNA and protein in the same cell) now allow high-resolution mapping of these regulatory mechanisms within heterogeneous tumor populations [61]. These approaches can identify rare resistant subclones governed by distinct post-transcriptional programs and clarify spatially resolved regulatory events in the TME. For example, a machine learning (ML)-guided single-cell multi-omics study recently uncovered an immunosuppressive niche driven by GDF15 in NSCLC, proposing a new framework for understanding and overcoming anti-PD-1 resistance [160]. This finding opens novel avenues for countering NSCLC drug resistance.

### Other omics combinations​

In researching drug resistance mechanisms in NSCLC, the limitations of single-omics technologies have become increasingly evident. Integrated multi-omics analysis has emerged as a crucial strategy for elucidating complex biological processes. The systematic integration of various omics layers—such as metabolomics, epigenomics, and proteomics—enables a multidimensional analysis of tumor drug resistance, providing a foundation for developing improved therapeutic strategies.

#### Integration of metabolomics and proteomics and its significance

The integration of metabolomics and proteomics offers powerful insights into the functional phenotypic changes underlying drug resistance by linking protein expression with metabolic rewiring. Metabolic reprogramming is a hallmark of drug-resistant NSCLC cells [27], and integrated analyses reveal how proteomic alterations drive these adaptive responses. For example, resistant cells exhibit upregulated expression of glycolytic enzymes such as hexokinase 2 (HK2) and pyruvate kinase M2 (PKM2). Correlative metabolomic profiling shows corresponding accumulations of lactate and glycolytic intermediates, indicating enhanced glycolytic flux that supports proliferation and resistance [18, 153].

This synergistic approach extends to other metabolic pathways. Increased abundance of glutamine-metabolizing enzymes detected via proteomics coincides with elevated levels of metabolites such as α-ketoglutarate, reflecting augmented anaplerosis and biosynthesis [24, 54, 150]. Similarly, proteomic identification of elevated fatty acid synthase aligns with metabolomic observations of increased lipid species, confirming enhanced lipogenesis that promotes membrane integrity and chemoresistance [17, 95, 123]. Notably, proteomic studies have identified key proteins that serve as biomarkers and functional drivers of resistance. For instance, overexpression of the transcriptional coactivator PGC-1α in resistant cells enhances mitochondrial metabolism and oxidative phosphorylation—a finding corroborated by metabolomic studies showing altered TCA cycle activity. This PGC-1α-mediated metabolic adaptation is proposed to enhance cell survival and contribute significantly to chemoresistance [59]. Proteomic analyses have also revealed biomarkers predictive of chemotherapy response. High expression of ERCC1, a nucleotide excision repair protein, is strongly associated with platinum resistance [71, 113], while overexpression of βIII-tubulin—detected proteomically—alters microtubule dynamics and reduces paclitaxel binding, serving as a validated biomarker of taxane resistance [45]. By integrating metabolomic and proteomic datasets, researchers can identify key regulatory nodes in resistance pathways and uncover actionable therapeutic vulnerabilities, thereby providing a comprehensive basis for targeting metabolic adaptations in resistant NSCLC.

#### The interaction between epigenomics and proteomics in drug resistance

Epigenomics involves the study of heritable modifications such as DNA methylation and histone modifications, that regulate gene expression without altering the DNA sequence. Proteomics reflects the functional outcomes of these epigenetic changes through corresponding protein expression dynamics [4, 91]. Integrating these two fields enables a thorough analysis of the dynamic interplay between epigenetic regulation and protein-level changes during NSCLC drug resistance.

At the DNA methylation level, studies have identified hypermethylation in the promoter regions of tumor suppressor genes in drug-resistant NSCLC cells. For example, epigenomic profiling reveals methylation of the RASSF1A promoter region, leading to transcriptional silencing. Reduced expression of RASSF1A protein—detected via proteomics—disrupts cell cycle regulation and apoptotic signaling, thereby promoting drug resistance [129, 136]. Histone modifications also contribute significantly to resistance. Changes in histone H3 lysine 27 trimethylation (H3K27me3) can modulate the expression of resistance-related genes. Epigenomic mapping of aberrant H3K27me3 enrichment, combined with proteomic analysis, has shown altered expression of encoded proteins—such as multidrug resistance-associated proteins—that influence drug transport and intracellular concentrations, ultimately leading to resistance [21, 36, 157, 158].

Furthermore, a feedback loop exists between epigenetic regulators and proteins. Altered expression of proteins such as transcription factors and epigenetic enzymes can, in turn, influence epigenetic landscapes. For instance, upregulation of DNA methyltransferases (DNMTs) in resistant NSCLC cells promotes further gene methylation, forming a self-reinforcing loop that maintains resistant phenotypes [2, 19–21]. Elucidating the interactions between epigenomics and proteomics provides new therapeutic targets and strategies.

## From drug target discovery to reversal strategies​

### Identification of novel drug targets through multi-omics​

Multi-omics integration provides a powerful strategy for the systematic discovery of novel drug targets. Through in-depth analysis of proteomic and other omics data, researchers can holistically and accurately identify potential therapeutic targets involved in NSCLC drug resistance.

#### Validating potential drug targets discovered from proteomics and multi-omics data

Integrated analysis of proteomic, transcriptomic, metabolomic, and epigenomic data can identify numerous candidate targets associated with NSCLC drug resistance [128, 134, 151, 157, 158]. However, these targets must undergo rigorous validation to confirm their suitability for therapeutic intervention. Initial validation typically employs molecular biology techniques such as RNA interference (RNAi) or gene knockout in NSCLC cell lines to assess changes in drug-resistant phenotypes following target inhibition. For example, the observation that RNAi-mediated knockdown of a gene (which encodes a protein overexpressed in resistance) restores cellular sensitivity suggests a functional role for this target in resistance mechanisms [72]. Conversely, overexpression of the candidate gene in drug-sensitive cells can be used to confirm induction of resistance, further supporting its importance. Animal models represent another critical step in target validation. Mouse models bearing human NSCLC-resistant tumors enable in vivo evaluation of target inhibition effects on tumor growth, metastasis, and treatment response, providing important evidence of efficacy and safety [26].

#### Prioritizing targets for drug development

Given the abundance of targets emerging from multi-omics studies, systematic prioritization is essential to focus development efforts. Key criteria include the biological importance of the target, prioritizing those that act at central nodes of critical signaling pathways that decisively influence tumor cell survival and resistance. For instance, in EGFR-TKI-resistant NSCLC, key proteins within activated bypass pathways such as MET signaling represent high-priority targets due to their critical role in maintaining the resistant state [102, 130].

Druggability is another essential factor, refers to the feasibility of targeting it with drugs, typically small molecules or antibodies [66]. Structural analysis helps determine whether the target contains tractable binding sites or epitopes, thereby influencing the likelihood of successful drug development. Additionally, targets with highly specific expression in tumors compared to normal tissues should be prioritized to minimize potential on-target toxicities [132, 145].

Furthermore, clinical unmet needs and limitations of existing therapies should guide prioritization. Targets associated with resistant subtypes or refractory NSCLC lacking effective treatments warrant higher priority. Through this type of multidimensional assessment, the most promising targets can be selected, accelerating the development of novel anticancer drugs and offering new therapeutic avenues for overcoming NSCLC drug resistance [86].

### Reversal strategies for NSCLC drug resistance​

Drug resistance remains a major constraint on clinical efficacy in NSCLC treatment. In response, researchers have developed various strategies to counteract resistance, informed by growing understanding of resistance mechanisms. Among these, small-molecule inhibitors targeting resistance-associated proteins and multi-omics-guided combination therapies represent particularly promising approaches.

#### Small molecule inhibitors targeting resistance-related proteins

Small-molecule inhibitors can specifically bind to and inhibit proteins implicated in drug resistance, thereby restoring tumor cell sensitivity. In the context of resistance to EGFR-TKIs, third-generation agents such as osimertinib serve as a prime example. Osimertinib selectively targets EGFR harboring the T790M mutation, suppressing its aberrant kinase activity, reversing resistance, and significantly prolonging patient survival [29].

Small-molecule inhibitors addressing other resistance mechanisms are also emerging. For example, in cases of multidrug resistance mediated by ABC transporters, inhibitors such as verapamil can competitively bind to these transporters and block efflux in preclinical models, although their clinical translation has been limited by toxicity and lack of specificity. For metabolic reprogramming in resistant tumors, inhibitors targeting key enzymes such as hexokinase 2 (HK2) can disrupt hyperactive glycolytic pathways, impair energy production, and help overcome resistance [6, 51, 154].

#### Combination therapies based on multi-omics insights​

Multi-omics integration provides a scientific foundation for designing rational combination therapies aimed at reversing NSCLC drug resistance. By capturing complex, overlapping resistance mechanisms through genomics, transcriptomics, proteomics, and metabolomics, researchers can identify co-activated pathways and design more effective treatment regimens [164].

In targeted therapy resistance, multi-omics analyses have revealed that resistance to EGFR-TKIs frequently involves bypass activation of pathways such as MET amplification or HER2 activation. Consequently, combination regimens integrating EGFR-TKIs with MET inhibitors (e.g., crizotinib) or HER2 inhibitors (e.g., trastuzumab) can simultaneously block multiple signaling axes, effectively suppressing tumor growth [39, 148]. In chemotherapy resistance, multi-omics studies have highlighted the role of the TME. Combining chemotherapy with agents targeting the TME—such as vascular endothelial growth factor (VEGF) inhibitors—can not only directly kill tumor cells but also modulate stromal interactions, reduce protective niche signals, and mitigate resistance [14, 42].

Moreover, multi-omics profiling aids in identifying biomarkers suitable for combination therapy, enabling clinicians to tailor regimens. This approach supports more precise and effective personalized treatment, offering new therapeutic opportunities for patients with resistant NSCLC [128, 134, 162].

## Challenges and future perspectives​

### Technical challenges in multi-omics

Although multi-omics technologies have significantly advanced research, they still face considerable technical challenges. Key limitations include difficulties in data integration and standardization, as well as the high cost and low throughput of certain technologies, which hinder large-scale and in-depth studies.

#### Data integration and normalization issues

Multi-omics research involves various types of data, such as genomics, transcriptomics, proteomics, and metabolomics. Each type of data has distinct formats, units, and analytical methods, making integration highly challenging [75]. For instance, transcriptomic RNA-seq data are quantified in expression values (e.g., FPKM, TPM), while proteomic mass spectrometry data reflect peptide intensities or protein abundances. These differences in dimensionality and statistical approaches complicate direct correlation analysis [14]. Additionally, systematic variations arise across experimental platforms and laboratories,even the same sample analyzed on different instruments may yield significantly divergent results [50]. Therefore, establishing unified data standards and effective normalization methods is crucial. Although standardized tools exist for individual omics data types (e.g., DESeq2 for transcriptomics, MaxQuant for proteomics), the integration and joint normalization of multi-omics data still lack universal and efficient solutions, which affects the accuracy and reliability of integrated analyses [115, 155]. To address these challenges, emerging computational strategies are being developed. AI-driven tools such as DeepOmics (a CNN-based framework) can automatically normalize heterogeneous data by learning cross-modality features, showing promise in reducing platform-specific biases in cancer datasets [109, 161]. Automated ML (AutoML) approaches help select optimal normalization strategies for combined genomics-proteomics data, better preserving biological variation than conventional methods [107]. Network-based frameworks also show promise: WGCNA constructs cross-omics co-expression networks to identify consensus modules (e.g., mRNA-protein clusters linked to EGFR mutations) [65], and MOFA decomposes multi-omics data into shared latent factors, revealing cross-layer regulatory axes (e.g., metabolomic-epigenomic interactions in drug resistance) [7]. These tools offer practical paths to mitigate integration barriers, though NSCLC-specific optimization (e.g., accounting for tumor heterogeneity) is still needed.

#### High-cost and low-throughput of some techniques

The high expense and limited throughput of certain multi-omics technologies constrain their large-scale application. For example, high-resolution mass spectrometers used in proteomics represent a significant capital investment, with additional ongoing expenses for reagents and maintenance, making them prohibitively expensive for many research institutions. Moreover, mass spectrometry sample preparation is complex and time-consuming, typically allowing only a limited number of samples per run—far fewer than high-throughput sequencing technologies [56]. These limitations of cost and throughput impede large-scale clinical cohort studies and rapid biomarker screening, posing a major barrier to the advancement and clinical translation of multi-omics research in NSCLC drug resistance.

### Translational challenges​

Although basic research ​​leveraging​​ advanced technologies has uncovered ​​multiple​​ resistance mechanisms, biomarkers, and therapeutic targets, translating these ​​discoveries​​ into clinically ​​actionable​​ solutions remains a significant challenge [55]. On one hand, basic research is typically conducted in highly ​​controlled​​ laboratory settings using cell lines and animal models that may not fully recapitulate the complexity of human tumors. Proteins or genes identified as drivers of resistance in preclinical models may not ​​function equivalently​​ in patients, owing to factors such as the TME and individual genetic variability [159]. This ​​disconnect​​ complicates direct translation to clinical practice. On the other hand, clinical validation is a protracted and intricate process. Biomarkers or targets identified in basic research must be validated in large-scale clinical cohorts, which demands substantial resources, extended timeframes, and coordinated efforts across multiple stakeholders. Challenges such as patient recruitment difficulties, incomplete sample collection, and inter-institutional variability often arise. For example, some resistance-associated proteins show promising predictive value in small studies but may exhibit reduced accuracy in larger, multicenter trials due to differences in patient demographics, disease characteristics, or treatment protocols [90]. Inadequate collaboration between basic researchers and clinicians further impedes translational progress. Disparities in research focus, expertise, and communication channels impede the alignment of research outcomes with clinical priorities. Furthermore, practical challenges encountered in clinical settings are frequently not effectively ​​fed back​​ to researchers, which slows iterative refinement and real-world application [3].

### Future directions

As advancements in life sciences continue to unfold, ​​single-omics approaches are proving increasingly insufficient​​ to address complex biological problems such as NSCLC drug resistance. Thus, the development of ​​more integrative​​ multi-omics platforms has emerged as a critical future direction. Currently, genomics, transcriptomics, proteomics, and metabolomics are frequently conducted in isolation, leading to ​​temporal inconsistencies​​ and analytical barriers in data integration [35]. Future efforts should prioritize enabling ​​simultaneous multi-omics profiling​​ from the same sample to reduce variability, enhance data consistency, and strengthen biological relevance. Single-cell multi-omics technologies—capable of capturing genomic, transcriptomic, and proteomic information from individual cells—will be particularly valuable for deciphering tumor heterogeneity and resistance mechanisms.

Enhancing the automation and intelligence of multi-omics platforms is also essential. The vast and complex datasets generated ​​demand​​ efficient processing beyond manual capabilities. AI and ML algorithms can ​​enable​​ automated data integration and analysis systems [104, 143], facilitating the rapid identification of key patterns and supporting more efficient discovery of resistance mechanisms and therapeutic targets [118]. Standardization is another critical factor. Establishing uniform protocols for data generation, storage, and analysis will ​​promote​​ cross-institutional data sharing and reproducibility [124]. A practical step could involve forming an International NSCLC Multi-Omics Standardization Committee—comprising researchers, clinicians, and bioinformaticians—to develop guidelines, organize inter-laboratory validations, and advance tool standardization (e.g., unified normalization software for proteomic-transcriptomic data).

Multi-omics integration also opens new avenues for personalized medicine in NSCLC. Each patient’s tumor harbors unique molecular features across genetic, transcriptional, proteomic, and metabolic levels. Multi-omics profiling can precisely characterize these traits, informing customized treatment strategies. Genomic data can identify actionable mutations; transcriptomic and proteomic data can reveal dysregulated pathways; and metabolomic data can uncover metabolic vulnerabilities [127]. Integrating this information would allow clinicians to tailor combinations of targeted therapy, immunotherapy, or chemotherapy to individual patients. Moreover, multi-omics-based predictive models could forecast treatment response and prognosis, helping clinicians select optimal therapies proactively, minimize adverse effects, and ​​advance​​ personalized treatment, ultimately improving both quality of life and survival outcomes for NSCLC patients [97].

## Conclusion​

In drug-resistant NSCLC, integration multi-omics approaches have revealed critical mechanisms like metabolic reprogramming. Similarly, ​​integrating​​ epigenomics with proteomics has demonstrated how regulatory changes (e.g., DNA methylation) influence the expression of resistance-related genes. These technologies ​​have facilitated​​ the discovery of novel therapeutic targets and strategies, including small-molecule inhibitors and immunotherapies, ​​and have supported​​ personalized treatment by improving prognostic accuracy and clinical decision-making.

However, multi-omics research still faces challenges such as ​​complexities in data integration​​, insufficient standardization, high costs, and low throughput of certain technologies. Moving forward, efforts should prioritize developing more ​​integrative​​ and automated multi-omics platforms, advancing AI-driven data analysis, establishing cross-institutional standardized protocols, and enhancing clinical translation via collaborative research. These advancements will be pivotal in driving the development of personalized medicine for NSCLC and overcoming drug resistance.

## Data Availability

No datasets were generated or analysed during the current study.
